# Recent Advances in the Photoautotrophic Metabolism of Cyanobacteria: Biotechnological Implications

**DOI:** 10.3390/life10050071

**Published:** 2020-05-19

**Authors:** Théo Veaudor, Victoire Blanc-Garin, Célia Chenebault, Encarnación Diaz-Santos, Jean-François Sassi, Corinne Cassier-Chauvat, Franck Chauvat

**Affiliations:** 1 Institute for Integrative Biology of the Cell (I2BC), Université Paris-Saclay, CEA, CNRS, 91198 Gif-sur-Yvette, France; theo.veaudor@cea.fr (T.V.); victoire.blanc-garin@cea.fr (V.B.-G.); celia.chenebault@cea.fr (C.C.); encdiasan@hotmail.com (E.D.-S.); corinne.cassier-chauvat@cea.fr (C.C.-C.); 2 Commissariat à l’énergie atomique et aux énergies alternatives (CEA), Centre de Cadarache St Paul Lez, 13108 Durance, France; Jean-Francois.SASSI@cea.fr

**Keywords:** cyanobacteria, biodiversity, photosynthesis, CO_2_ assimilation, nitrogen fixation, metabolic plasticity, regulation, redox control, production of chemicals for health and industries

## Abstract

Cyanobacteria constitute the only phylum of oxygen-evolving photosynthetic prokaryotes that shaped the oxygenic atmosphere of our planet. Over time, cyanobacteria have evolved as a widely diverse group of organisms that have colonized most aquatic and soil ecosystems of our planet and constitute a large proportion of the biomass that sustains the biosphere. Cyanobacteria synthesize a vast array of biologically active metabolites that are of great interest for human health and industry, and several model cyanobacteria can be genetically manipulated. Hence, cyanobacteria are regarded as promising microbial factories for the production of chemicals from highly abundant natural resources, e.g., solar energy, CO_2_, minerals, and waters, eventually coupled to wastewater treatment to save costs. In this review, we summarize new important discoveries on the plasticity of the photoautotrophic metabolism of cyanobacteria, emphasizing the coordinated partitioning of carbon and nitrogen towards growth or compound storage, and the importance of these processes for biotechnological perspectives. We also emphasize the importance of redox regulation (including glutathionylation) on these processes, a subject which has often been overlooked.

## 1. Introduction

Cyanobacteria are very ancient organisms (from 2.3 to 3.5 billion years old [1]) that perform oxygen-evolving (plant-like) photosynthesis. These organisms are regarded as the ancestors of the plant chloroplast [2] and were responsible for oxygenation of the Earth’s atmosphere [3]. Present-day cyanobacteria are responsible for ~25% of the organic carbon fixation on Earth [4], and some strains that can also fix atmospheric nitrogen (N_2_) have been used for a long time for the nitrogen fertilization of rice fields [5]. Cyanobacteria are surrounded by a gram-negative cell envelope that comprises an outer membrane, a peptidoglycan layer and a plasma membrane [6]. They also possess thylakoids, the internal membranes that contain both the photosynthetic and respiratory machineries, excepting strains of the genus *Gloeobacter* that possess only the cytoplasmic membrane [7]. 

In colonizing most aquatic ecosystems (fresh, brackish, and marine) and soils (including deserts) of our planet, where they face environmental challenges, cyanobacteria have evolved as a widely diverse group of organisms. They display various cell morphologies [8], and widely diverse genome sizes (from 1.44 to 12.07 Mb) and organization [9]. All cyanobacteria possess a circular chromosome and, depending on the particular strain, ecotype or species, a linear chromosome (rarely) and/or one to several circular plasmids (frequently).

Cyanobacteria produce a wealth of metabolites that can influence (i) their tolerance to environmental stresses; (ii) their interactions with competitors, predators or symbiotic hosts; and (iii) human health including: antioxidants, antibacterial, antifungal, antiviral, toxins, and vitamins [10,11,12]. Cyanobacteria of the genera *Arthrospira* (often misidentified as *Spirulina*), *Anabaena* and *Nostoc* have been consumed by humans for over a thousand years [13]. Currently, *Arthrospira platensis* and *Arthrospira maxima* serve as a highly digestible supplementary source of high-quality proteins, vitamins, minerals, and essential fatty acids. A few edible cyanobacteria are being tested as a way to replenish O_2_, provide food, and recycle CO_2_ and urea wastes during long-term space missions [14]. Furthermore, genome analyses have indicated that cyanobacteria have the potential to produce many more metabolites than those characterized so far [11,15]. Thus, it should be interesting in the future to exploit the metabolic diversity of cyanobacteria (they produce a wealth of natural products), their photosynthesis, and their radiation resistance (they can grow on ^14^CO_2_) for the production of ^14^C-labelled bioactive metabolites to screen for new drugs and analyze their absorption, distribution, metabolism, and excretion properties in mammalian cells and tissues [16]. 

Cyanobacteria are also increasingly regarded as promising cell factories for the production of high value-added chemicals (biofuels, bioplastic, cosmetics, drugs, and vitamins) from highly-abundant natural resources such as solar energy, water, CO_2_, and minerals [6,17,18], eventually being combined with wastewater treatment to reduce costs [5]. Cyanobacteria capture solar energy at high efficiencies (3–9%) [19] to fix annually about 25 gigatons of carbon from atmospheric CO_2_ into a huge energy-dense biomass [20], and they can also tolerate high CO_2_-containing (≥50%) industrial gas [19].

Furthermore, cyanobacteria also have the potential for the photoproduction of hydrogen (H_2_) that has a higher energy content than oil (142 MJ/kg for H2 vs. 44.2 MJ/kg for oil) and burns cleanly, producing only water as the by-product. Indeed, cyanobacteria possess the two enzymes that produce hydrogen, (bidirectional) hydrogenase [21] and nitrogenase [22]. However, as these two enzymes are sensitive to oxygen they need to be engineered to become oxygen tolerant [21,22]

In the following sections, the plasticity of the photoautotrophic metabolism of cyanobacteria will be placed in the context of the great interest in these organisms for basic and applied science objectives. We will emphasize the best-studied models *Synechocystis* PCC 6803, *Synechococcus* PCC 7942, and *Synechococcus* PCC 7002 because they possess a simple (unicellular) morphology, a small genome (3.95; 2.75, and 3.40 Mb, respectively) and powerful genetic tools [23]. Furthermore, these three cyanobacteria possess interesting physiological and metabolic differences. Both *Synechocystis* PCC 6803 and *Synechococcus* PCC 7002 are euryhaline cyanobacteria (*Synechococcus* PCC 7002 is a costal strain), and they are both able to grow on urea as the sole nitrogen source, unlike the freshwater organism *Synechococcus* PCC 7942 [24]. The growth of *Synechococcus* PCC 7002 requires vitamin B12 and it can be accelerated by glycerol [25], a cheap surplus of oil industries that can improve microbiological production [26]. In contrast, *Synechocystis* PCC 6803 and *Synechococcus* PCC 7942 do not need vitamin B12 and they cannot use glycerol for faster growth (it is even toxic for *Synechocystis* PCC 6803). Furthermore, *Synechocystis* PCC 6803 can grow on glucose under very-low light or darkness, unlike *Synechococcus* PCC 7942 and *Synechococcus* PCC 7002 [27].

## 2. Cyanobacteria Perform Oxygenic Photosynthesis to Power up Their Photoautotrophic Metabolism

Cyanobacteria capture light by their antenna that are composed of chlorophyll and phycobiliproteins, which covalently bind linear tetrapyrrole pigments called bilins as well as linker proteins. The phycobilisomes are assemblies of phycobiliproteins that have a core made up of allophycocyanin, from which phycocyanin rods project. In some cyanobacteria, the rods also contain phycoerythrin in addition to phycocyanin [28]. The light-harvesting antennae serve a dual role in photosynthesis, depending on the light intensities. Under "physiological" light, they ensure photosynthetic efficiency by maximizing the light absorption cross-section and subsequent energy storage. Under excess light conditions, the light-harvesting complexes perform photoprotective quenching functions to prevent harmful chemical species such as triplet chlorophyll and singlet oxygen from forming and damaging the photosynthetic apparatus [28].

Solar energy captured by the antenna is transferred to the multi-subunits photosystems II (PSII) that uses it to split H_2_O_2_ molecules into O_2_ and reducing equivalents. Four high energy electrons, together with four protons (4H^+^), are used to reduce plastoquinone (PQ), the terminal electron acceptor of PSII, to plastoquinol (PQH2). Then, PQH2 passes its reducing equivalents to an electron transfer chain, which feeds into photosystem I (PSI) where they gain additional reducing potential from a second light reaction [29], to generate chemical energy (ATP) and reducing power (NADPH) [30]. This chemical energy powers up the assimilation of inorganic nutrients, including carbon (CO_2_) nitrogen (N_2_, NO_3_, NH_4_) (see below), sulfur (SO_4_), and other metals and minerals (Ca, Cu, Fe, K, Mg, Mn, P, Zn, *etc*) to produce a huge biomass that sustains most life forms on Earth. The Great Oxidation Event (GOE) that occurred 2.4 billion years ago likely resulted from the accumulation of oxygen evolved by the ancestors of cyanobacteria [3]. Cyanobacteria can also assimilate organic compounds such as carbohydrates, amino acids, or urea [24,25,27].

Cyanobacteria perform both photosynthesis and respiration in cell and intra-cytoplasmic membranes. Photosynthesis takes place in the thylakoids, respiration occurs in the cytoplasmic and in the thylakoid membranes, excepting members of the *Gloeobacter* genus that have only the cytoplasmic membrane [7]. Some cyanobacteria living exclusively in sulfide-rich microbial mats can also switch between oxygenic and anoxygenic photosynthesis, which relies on PSI only [31]. In darkness, the regular mode of cyanobacterial metabolism is aerobic respiration, while cyanobacterial species that are often exposed to anaerobic conditions also have the capacity of fermenting internal carbon-storage compounds, e.g., glycogen (glycogen) [6]. Fermentation likely operates for the generation of energy to cope with long periods of darkness under anoxic conditions.

## 3. RubisCO: The Key Enzyme Player in CO_2_ Fixation

Carbon fixation is arguably one of the most important metabolic processes on Earth. A large part of the biological fixation of CO_2_ (260 gigatons tons, annually [32]) is carried out by the Calvin−Benson−Bassham metabolic pathway (CBB cycle, hereafter referred to as the Calvin cycle), which uses the ATP energy and NADPH reducing power provided by photosynthetic electron transport. The key ribulose bisphosphate carboxylase/oxygenase enzyme (RubisCO) catalyzes the addition of one molecule of CO_2_ to one molecule of the five-carbons sugar 1,5-ribulose bisphosphate (RuBP) to generate two molecules of the three-carbons 3-phosphoglycerate (3PGA). They then undergo a series of interconversions to form the six-carbons sugar fructose-1,6-bisphosphate (FBP), while regenerating RuBP. The net Calvin cycle reaction is given by the following equation

3CO_2_ + 5H_2_O + 6NADPH + 9ATP → 3PGA + 6NADP + 9ADP + 8 Pi. (1)

Considering its pivotal role, RubisCO is surprisingly inefficient as an enzyme [32,33]. In plants, its slow catalytic rate of only ~2–10 CO_2_ molecules fixed per second (compared to above 100 molecules of substrate per second for most enzymes) necessitates the production of significant levels of RubisCO, which can reach levels as high as 50% of the soluble leaf proteins [32]. Cyanobacterial RubisCO is a ~550-kDa hexadecameric complex consisting of eight large (RbcL, ~50–55 kDa) and eight small (RbcS, ~12–18 kDa) subunits. The assembly of the RbcL8S8 holoenzyme is thought to involve the formation of an RbcL8 core, followed by the docking of RbcS subunits. RubisCO assembly involves the ATP-independent chaperons RbcX along with Raf1 and Raf2, which also occur in plants and green algae. RbcX has been reported to be essential in *Synechococcus* PCC 7002, although not in *Synechocystis* PCC 6803 [34] nor in *Synechococcus* PCC 7942 [32]. Raf1 is present in all cyanobacteria that also have RbcX. Both RbcX and Raf1 stabilize the RbcL2 unit by using different interaction sites on RbcL [35]. Raf2 homologs have been identified in a subset of (marine) cyanobacteria expressing form IA RubisCO.

The RubisCO enzyme cannot discriminate O_2_ from CO_2_. Its photorespiration activity drives the oxygenation of RuBP that generates one molecule each of 3PGA and 2-phosphoglycolate (2PG). As 2PG is toxic, cyanobacteria have evolved strategies to recycle two molecules of 2PG to one molecule of 3PGA, while releasing CO_2_. Thus, 75% of (fixed) organic carbon is salvaged and used to synthesize RuBP and refill the Calvin cycle [36].

Several strategies are being used to increase the cyanobacterial RubisCO activity for biotechnological purposes, such as directed evolution, for example. This uses the generation of a library of mutants followed by a selection process, to identify mutant proteins with the desired properties. In one study, an engineered *E. coli* reporter strain dependent on a functional RubisCO for growth was developed to screen libraries of RubisCO mutants [32]. They all possessed an increased solubility, but not improved catalytic properties, though they did not show improved photosynthesis when tested in cyanobacteria [32]. Similarly, successive rounds of mutation and selection in *E. coli* of the RubisCO from the cyanobacterium *Thermosynechococcus elongatus* BP1 identified two RbcL and six RbcS mutations that improved the solubility and carboxylation efficiency of RubisCO: 3–14 and ~40 fold, respectively [37]. In another study, a strain of the purple bacterium *Rhodobacter capsulatus* lacking its own RubisCO was used as a reporter host for selecting variants of *Synechococcus* PCC 6301 RubisCO that provided a more active CO_2_-dependent growth than the wild-type enzyme [38]. One of these mutations improved (~2-fold) the carboxylase activity of RubisCO. In the other cases, the enhanced growth performance was traceable to differential interactions of the mutant enzymes with CO_2_ and O_2_. Effective amino acid substitutions also appeared to be localized to two other conserved hydrophobic regions of the holoenzyme. 

Instead of attempting to increase the activity of RubisCO, overexpression and FLAG-tagging RubisCO in engineered *Synechocystis* PCC 6803 have helped to increase the abundance of RubisCO and enhance (~2-fold) CO_2_ fixation [39]. Furthermore, the overproduction of any of the Calvin cycle enzymes, RuBisCO, sedoheptulose bisphosphatase, fructose bisphosphate aldolase, or transketolase, improved (~50%) the total carbon fixation rates in *Synechocystis* PCC 6803 [40]. Interestingly, from a biotechnological perspective, the cloning of additional RubisCO genes from *Synechococcus* PCC 6301 in *Synechococcus* PCC 7942 expressing heterologous isobutyraldehyde synthesis genes improved RubisCO activity (~1.5-fold) and the production (~2-fold) of isobutyraldehyde [41]. Similarly, a three-fold increased fatty acid production by *Synechococcus* PCC 7002 was observed after the overproduction of the *Synechococcus* PCC 7942 RuBisCO [42]. 

Exogenous pathways can also be integrated into cyanobacteria to decrease the undesired photorespiration that produces the toxic metabolite 2PG. Heterologous expression of an oxygen-insensitive 3-hydroxyproprionate bi-cycle in *Synechococcus* PCC 7942 has been able to reduce the synthesis of 2PG [43]. This six-enzymes cycle was designed to function as both a photorespiratory bypass and an additional CO_2_-fixing pathway, supplementing the Calvin cycle. This synthetic enzyme system, in producing a phosphoglycerate phosphatase and a glycolate dehydrogenase, redirects glyoxylate, originating from 2PG, toward the synthesis of pyruvate and it fixes an additional two molecules of HCO_3_^−^ [39,43]. 

## 4. Carboxysome A Complex Intracellular Compartment for CO_2_ Fixation

**Figure 1:**
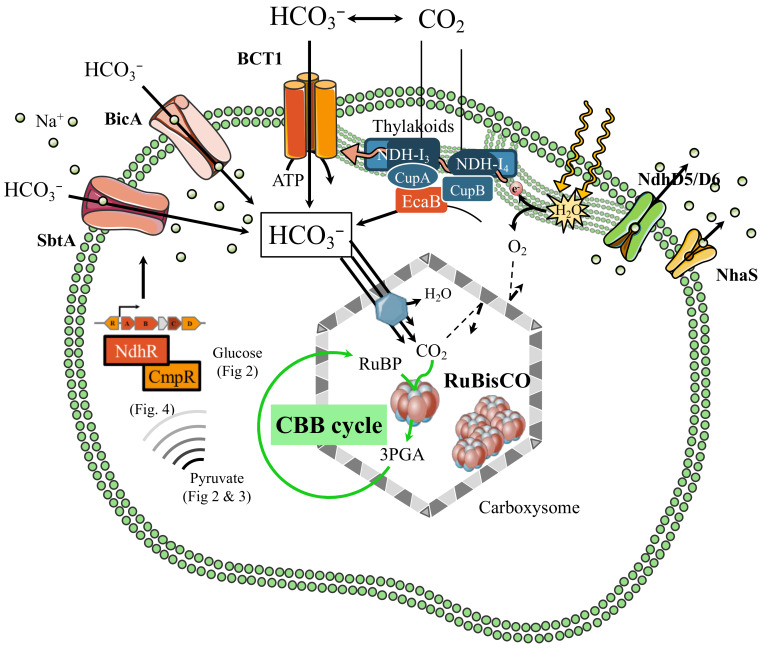
Schematic representation of the carbon concentrating mechanism in *Synechocystis* PCC 6803. It comprises two CO_2_ uptake systems, a Na^+^/H^+^ gradient-restoring system, and three HCO_3_^−^ transporters, as well as the carboxysome. Inside the carboxysome, the carbonic anhydrase (CA) enzyme dehydrates HCO_3_^−^ to CO_2_, that is subsequently fixed by RubisCO (RuBP (ribulose-1,5-bisphosphate) + CO_2_ to yield 2 molecules of 3PGA (3-phosphoglycerate). The triose phosphates derived from 3PGA can be used by the Calvin cycle to regenerate RuBP or be further catabolized (see Figure 2 and Figure 3). Some intermediates of these pathways serve as metabolic signals and effectors for transcriptional regulation of the carbon concentrating mechanism (CCM) described (see Figure 4). The two thylakoid-bound CO_2_ uptake systems are: (i) the low-flux/high-affinity/low Ci-inducible NDH-I_3_ complex consisting of the NdhF3/NdhD3/CupA proteins and (ii), the high-flux/low-affinity/constitutive complex NDH-I_4_ comprising the NdhF4/NdhD4/CupB subunits. The NDH-I_3/4_ complexes possess CA-like activity driven by the CupA/CupB-interacting CA enzyme EcaB, which converts CO_2_ into HCO_3_^−^ by creating a proton gradient. The HCO_3_^−^ uptake systems consist of: (i) the high-affinity ATP-consuming (ABC-type) BCT1 transporter (comprising the CmpA/B/C/D subunits) and two sodium-dependent transporters: SbtA (low-flux/high-affinity/low-Ci-inducible) and a high-flux/medium-affinity transporter, BicA. NhaS or other subunits of NDH-1 complexes located within the membrane (NdhD5/D6) export sodium to balance its intracellular concentration.

To favor the carbon-fixing (carboxylase) activity of RubisCO over its oxygenase activity, cyanobacteria have evolved a carbon concentrating mechanism (CCM, Figure 1), which uses bicarbonate transporters to actively transport bicarbonate into the cell to overcome the slower (10^4^-fold) diffusion rates of CO_2_ in water compared to air [23]. An important part of the CCM is the subcellular compartment, called the carboxysome, that encapsulates the RubisCO and carbonic anhydrase enzymes in a CO_2_ concentrating environment [44]. The carboxysome, likely assembled around RubisCO [4], comprises hundreds of protein homologs that self-assemble in space to form an icosahedral structure [36]. The carboxysome shell favors the selective entry of the charged molecules of RuBP and bicarbonate (HCO_3_^−^), over the uncharged molecules of O_2_ and CO_2_. The diffusion of CO_2_ across the carboxysome (10^−5^ cm·s^−1^) is significantly slower than CO_2_ crossing the cellular membrane (10^−2^ cm/s) [45]. Following this, the carbonic anhydrase (CA) catalyzes the hydration of CO_2_ and dehydration of HCO_3_^−^ inside the carboxysome lumen to accumulate CO_2_ in the vicinity of RubisCO, which fixes CO_2_ to generate 3-phosphoglycerate (3PGA) that then leaves the carboxysome and is transformed into sugars by the cytosolic Calvin-cycle enzymes [46].

Two types of carboxysomes can be distinguished depending on the nature of their encapsulated RubisCO [44]. The α-carboxysomes (containing RubisCO Form IA) occur in α-cyanobacteria (typically marine cyanobacteria), while β-carboxysomes (harboring RubisCO Form IB) are present in β-cyanobacteria (mostly freshwater cyanobacteria). In α-carboxysomes the CA activity is provided by a β-CA (CsoSCA) associated with the shell [47]; whereas in β-carboxysomes it is mostly a γ-CA (CcmM), which also interacts with the shell, or in some cases an ancillary β-CA (CcaA) [48,49].

*Synechococcus* PCC 7942 and *Synechocystis* PCC 6803 possess prokaryotic β-type CA encoded by the *icfA* and *ccaA* genes, respectively, which share over 55% sequence identity and harbor a 50–60 amino acids C-terminus essential to localizing CAs near RubisCO within a carboxysome. 

In biological systems, where inorganic carbon can exist in the forms of H_2_CO_3_, HCO_3_^−^, and CO_3_^−2^, the pH affects the CCM efficiency and increases the selectivity of RubisCO toward CO_2_ over O_2_. At pH = 7.5, the carboxylase activity of RubisCO is maximal. At higher pH ranges between 7.5 and 8.5, the reduced energetic costs of HCO_3_^−^ transportation into the cytosol reduce the carbon fixation cost [39]. This pH range is within the natural pH levels of *Synechococcus* PCC 7942 incubated in darkness (7.3) and light (8.4) [50].

The essential genes for α- and β-carboxysomes are encoded by the *cso* and the *ccm* operons, respectively, which are nested within conserved gene clusters that also encode Ci transporters and other ancillary proteins [48]. 

The β-carboxysomes of *Synechococcus* PCC 7942 have been extensively characterized. They are composed of: (i) the hexameric proteins CcmK2, CcmK3, and CcmK4 forming the shell facets; (ii) the pentameric protein CcmL capping the vertices of the polyhedron; and (iii) the trimeric proteins CcmO and CcmP. The core enzymes of β-carboxysomes consist of a paracrystalline arrangement of RubisCO (comprising the large and small subunits RbcL and RbcS assembled in a RbcL8S8 holoenzyme) and β-carbonic anhydrase (CcaA). In addition, the “linker” proteins CcmM and CcmN promote RubisCO packing and shell-interior association. CcmM occurs as two isoforms, the 58-kD full-length form and a 35-kD truncated version. CcmM58, which recruits CcaA to the shell, has a N-terminal α-CA-like domain and the three RbcS-like domains which interact with RubisCO and are also present also in CcmM35 [49]. In addition, RbcX, a RubisCO chaperonin, performs a role in carboxysome assembly and sub-cellular distribution [51]. Interestingly, RbcX was found to be essential in *Synechococcus* PCC 7002, but not in *Synechococcus* PCC 7942 [52].

### 4.1. Carboxysomes Stochiometry

Recently, live-cell single-molecule fluorescence microscopy, coupled with confocal and electron microscopy, were used to decipher the protein stoichiometry, diameter, localization, and mobility patterns of single β-carboxysomes in *Synechococcus* PCC 7942. These parameters appeared to depend on CO_2_ levels and light intensity occurring during cell growth [51]. It has been estimated that there are about 1500, 850, 550, and 370 copies of RubisCO per β-carboxysome under high light (80 µE m^−2^·s^−1^), moderate light (ML, 50 µE m^−2^·s^−1^), ML + high CO_2_ (3% v/v), and low light (LL, 10 µE m^-2^·s^−1^), respectively. This study also confirmed the different interior organization of β-carboxysomes (densely packed with RubisCO forming paracrystalline arrays) as compared to α-carboxysomes (random packing of RubisCO). These differences may result from the distinct carboxysome biogenesis pathways; assembly of β-carboxysomes is initiated by the nucleation of RubisCO and CcmM35 that precedes shell encapsulation, whereas α-carboxysome biogenesis starts from shell formation and/or a shell-interior assembly [53].

These findings will certainly influence the rational design and construction of cyanobacterial cell factories for the photosynthetic production of chemicals, as well as the installation of powerful carboxysomes into plants, especially C_3_ plants, to increase the photosynthetic fixation of CO_2_ and improve crop production [51]. 

### 4.2. Carboxysome Positioning Systems 

The ability for cells to organize their interior space is ubiquitous among all organisms. In cyanobacteria [39], each daughter cell receives an equal number of carboxysomes during cell division thanks to the two-component McdAB system that equidistantly positions each carboxysome relative to the others [54]. 

Hence, in *Synechococcus* PCC 7942 approximately four carboxysomes are evenly spaced in the cytosol along the length of each rod-shaped cell. McdA, a ParA-type ATPase, non-specifically binds the nucleoid in the presence of ATP, while McdB directly binds carboxysomes, and displaces McdA from the nucleoid. Thus, *parA* deletion strains have unevenly spaced carboxysomes [54]. The McdAB system, absent in α-cyanobacteria, is widespread among β-cyanobacteria, with the type 2 McdAB system being the most ancestral and frequent, while the type 1 system, like that of *Synechococcus* PCC 7942, appears to be of more recent origin. These two McdA types may also have differences in ATPase activity, as suggested by their differences in amino acid sequence in and around the ATP-binding motifs. McdAB positioning of carboxysomes also operates in spherical-celled cyanobacterial, whereas no *mcdAB* genes were observed in many cyanobacteria displaying baeocystous morphologies [54]. 

Together, the above-mentioned conceptual frameworks provide the foundation for ‘plug-and-play’ engineering of carboxysomes as CO_2_ fixation modules in a variety of biotechnological applications (production of chemicals from solar energy and CO_2_). 

## 5. Phosphoenol-Pyruvate-Carboxylase: Another Important Player in CO_2_ Fixation

In addition to RubisCO, the phosphoenolpyruvate carboxylase (PEPC) enzyme catalyzes an irreversible carboxylation of the three-carbons metabolite phosphoenolpyruvate (PEP) with bicarbonate (HCO_3_^−^) to generate oxaloacetate (a four-carbons metabolite of the citric acid cycle) and inorganic phosphate. PEPC, which is widely distributed in cyanobacteria, was shown to account for 25% of CO_2_ fixation in *Synechocystis* PCC 6803 [55], and to be essential for the photoautotrophic growth of this organism [56] and *Synechococcus* PCC 7942 [57].

Attesting to the importance of PEPC for biotechnological work, it was shown in *Synechocystis* PCC 6803 that the overproduction of PEPC increased the production of chemicals such as succinate [58] and ethylene [59]. As well, the downregulation of pyruvate dehydrogenase (PDH) and PEP-C provided significant improvements in aldehyde production pathway by *Synechococcus* PCC 7942 [60]. 

## 6. Plasticity of the Cyanobacterial Carbon Metabolism

As is true for all organisms, the metabolism of cyanobacteria is tightly linked with, and capable of responding to, environmental conditions.

### 6.1. Occurrence of Multiple Glycolytic Pathways

Cyanobacteria possess all known glycolytic variant pathways: The Embden–Meyerhof–Parnas (EMP) pathway, the oxidative pentose phosphate (OPP) pathway, the phosphoketolase (PK) pathway, and the Entner–Doudoroff (ED) pathway [61]. They all share reactions and metabolites with the Calvin cycle and provide ATP, NAD(P)H, and carbon precursors for the synthesis of amino acids, nucleotides, and fatty acids [61,62]. 

**Figure 2:**
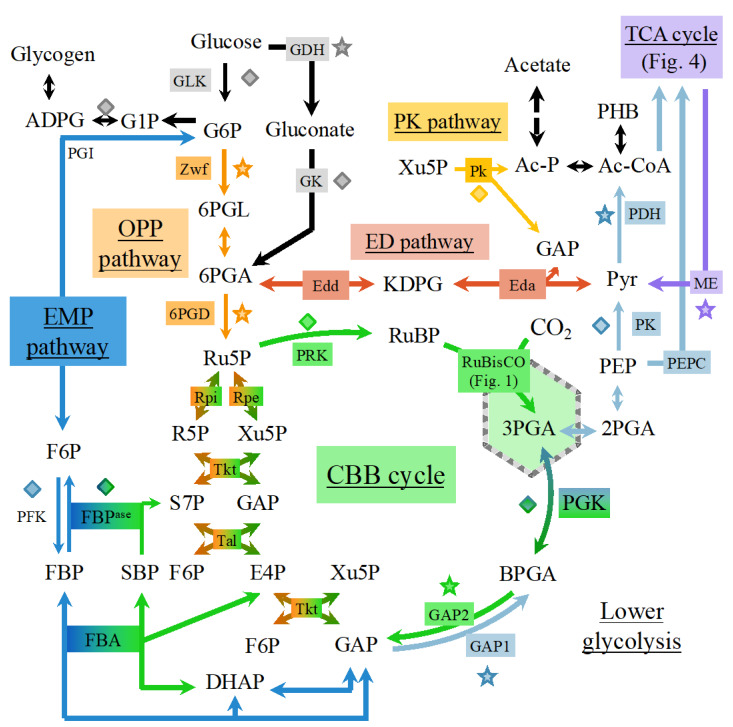
Schematic representation of the carbon metabolism in *Synechocystis* PCC 6803. These pathways comprise the EMP pathway (Embden–Meyerhof–Parnas, upper glycolysis and neoglucogenesis, typical reactions in dark blue), lower glycolysis (light blue), the ED pathway (Entner-Doudoroff; red), the OPP pathway (oxidative pentose phosphate; orange), the CBB cycle (Calvin-Benson-Bassham; green) and the recently discovered phosphoketolase pathway (yellow). Diamonds and stars indicate reactions using ATP or electron carriers (mostly NADPH and NADH) as cofactors, respectively. ADPG: ADP-glucose; BPGA: bisphosphoglycerate; Ac-CoA: acetyl-CoA; Ac-P: acetyl phosphate; DHAP: dihydroxyacetone phosphate; E4P: erythrose-4-phosphate; Edda: KDPG aldolase; Edd: 6PGA dehydratase; FBA: fructose-1,6-bisphosphate aldolase; FBP^ase^: fructose-1,6-bisphosphate; F6P: fructose-6-phosphate; G1P: glucose-1-phosphate; G6P: glucose-6-phosphate; GAP: glyceraldehyde-3-phosphate; GAP1-2: glyceraldehyde-3-phosphate dehydrogenase 1 or 2; GDH: glucose dehydrogenase; GK: gluconate kinase; GLK: glucokinase; KDPG: keto-3-deoxy-6-phospho-gluconate; ME: malic enzyme; PDH: pyruvate dehydrogenase; PEP: phosphoenolpyruvate; PEPC: PEP carboxylase; PFK: phosphofructokinase; PGI: phosphoglucose isomerase; PGK: phosphoglycerate kinase; PHB: poly-β-hydroxybutyrate; Pk: phosphoketolase; PK: pyruvate kinase; PRK: phosphoribulokinase; Pyr: pyruvate; Rpe: Ru5P epimerase; R5P: ribose-5-phosphate; Rpi: R5P isomerase; RuBisCO: ribulose-1,5-bisphosphate carboxylase/oxygenase; Ru5P: ribulose-5-phosphate; RuBP: ribulose-1,5-bisphosphate; S7P: sedoheptulose-7-phosphate; SBP: sedoheptulose-1,7-bisphosphate; Tal: transaldolase; Tkt: transketolase; Xu5P: Xylulose-5-phosphate; Zwf: glucose-6-phosphate dehydrogenase; 2PGA: 2-phosphoglycerate; 3PGA: 3-phosphoglycerate; 6PGL: 6-phosphogluconolactone; 6PGA: 6-phosphogluconate; 6PGD: 6-phosphogluconate dehydrogenase.

In the EMP pathway (Figure 2), glucose is phosphorylated twice to generate fructose bisphosphate (FBP) using the phosphofructokinase enzyme (PFK) that is unique to the EMP pathway. Then, fructose bisphosphate is cleaved into two triose-phosphates: glyceraldehyde-3-phosphate (GAP) and dihydroxyacetone phosphate (DHAP), which are both used to produce ATP through the phosphorylations occurring in lower glycolysis [61].

In the ED pathway (Figure 2), glucose is phosphorylated only once to glucose-6P (G6P) and sequentially converted to 6-phosphogluconate (6PGA) and 2-keto-3-deoxy-6-phospho-gluconate (KDPG), a metabolite unique to the ED pathway, which is then cleaved into one pyruvate and one GAP that is used to produce ATP. The ED pathway requires 3.5 times less enzymatic proteins to achieve the same glycolytic flux as the EMP pathways. However, one glucose molecule catabolized by EMP generates two ATP and two NADH molecules; whereas if one glucose molecule is catabolized by the ED pathway it yields one ATP, one NADH, and one NADPH. Usually cyanobacteria rely on the ED pathway rather than on the EMP pathway because they are nutrient-limited, rather than ATP-limited [61].

The OPP pathway (Figure 2) redirects 6PGA to pentose phosphates, generating an extra NADPH through 6PGA-dehydrogenase (6PGAD). Pentose phosphates are then converted to triose phosphates by the transketolase and transaldolase activities. As the pentose phosphate pathway can either run in its oxidative mode (OPP pathway) to oxidize carbohydrates or in its reductive mode (Calvin cycle) to fix CO_2_, it is tightly regulated [61].

The PK pathway (Figure 2) differs from the aforementioned pathways in splitting phosphorylated sugars (G6P/xylulose 5-phosphate) to produce C2 units (acetyl phosphate), thereby bypassing the oxidation of triose to acetyl compounds. 

The multiple glycolytic pathways of cyanobacteria differ in the number of ATP and NAD(P)H molecules they produce per molecule of glucose catabolized, ranging from 1 to 2.33 for ATP and 0 to 5.33 for NAD(P)H. These pathways allow cyanobacteria to promptly respond to variations in energy input from sunlight and/or organic compounds [62].

In *Synechocystis* PCC 6803, which can grow under photoautotrophic (light + CO_2_), mixotrophic (light + CO_2_ +glucose), and heterotrophic (glucose catabolism) conditions, the Calvin cycle, OPP, and EMP plastic pathways can regulate their fluxes under various growth conditions. In contrast, the (rigid) tricarboxylic acid cycle (TCA) always operates at relatively low levels. Under dark or low light conditions, the metabolism relies upon oxidative phosphorylation to generate ATP from NADPH. Moreover, a negligible flux was detected through the EDP pathway under heterotrophic conditions [63]. 

### 6.2. Occurrence of Multiple Tricarboxylic Acid Cycles 

**Figure 3:**
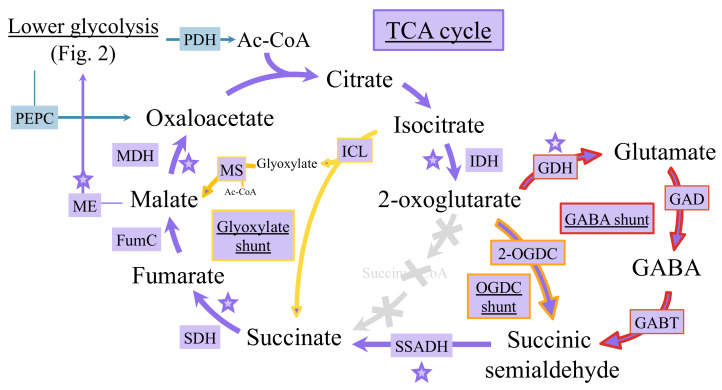
Schematic representation of the cyanobacterial shunts of the tricarboxylic acid (TCA) cycle. Cyanobacteria possess an atypical TCA cycle lacking the 2-oxoglutarate (2OG) dehydrogenase, which normally transforms 2-OG to succinyl-CoA that generates succinate via the succinate-CoA ligase. The gap in between 2OG and succinate can be filled by at least three different metabolic shunts. The glyoxylate shunt (outlined in yellow) produces succinate directly from isocitrate by isocitrate lyase. The 2OGDC shunt (outlined in orange) converts 2-oxoglutarate (2OG) to succinate via the 2OG decarboxylase (2OGDC) and succinic semialdehyde (SSA) dehydrogenase enzymes. The γ-aminobutyrate or GABA shunt (outlined in red) converts glutamate to succinate using the GABA amino-transferase and succinic semialdehyde (SSA) dehydrogenase enzymes. Diamonds and stars indicate reactions using ATP or electron carriers (generally NADPH and NADH) as cofactors, respectively. FumC: fumarase; GABT: GABA aminotransferase; GAD: glutamate decarboxylase; GDH: glutamate dehydrogenase; ICL: isocitrate lyase; IDH: isocitrate dehydrogenase; MDH: malate dehydrogenase; ME: malic enzyme; MS: malate synthase; PDH: pyruvate dehydrogenase; PEPC: PEP carboxylase; SDH: succinate dehydrogenase; SSADH: succinic semialdehyde dehydrogenase.

As observed for the glycolytic pathways (Figure 2), cyanobacteria possess more than one version of the TCA cycle that can synthesize important metabolites at the interface of carbon and nitrogen metabolisms [62]. The TCA cycle has long been considered as incomplete because of the lack of the 2-oxoglutarate dehydrogenase enzyme that normally converts 2-oxoglutarate (2OG, also called α-ketoglutarate) to succinyl-CoA, which is then transformed to succinate by the succinate-CoA ligase. Recently, it has been shown that the TCA gap between 2OG and succinate can be filled by at least three different metabolic shunts: the 2OG decarboxylase (OGDC) bypass, the gamma-aminobutyric acid (GABA) shunt, and the glyoxylate bypass (Figure 3).

The OGDC shunt converts 2OG to succinate via succinic semialdehyde (SSA), using OGDC and SSA dehydrogenase (SSADH) [64]. The sll1981 product from *Synechocystis* PCC 6803, previously annotated as L-myo-inositol-1-phosphate synthase, was shown to function as OGDC [36]. 

The GABA shunt connects 2OG and succinate, via glutamate decarboxylase (GAD), GABA aminotransferase, and SSADH, respectively [64]. In *Synechocystis* PCC 6803, which harbors the enzymes for both OGDC and GABA shunts, the GABA route was proposed to be more active than the OGDC route [36].

The glyoxylate shunt, which exists in very few genera of cyanobacteria (such as the two *Cyanothece* strains PCC 7424 and PCC 7822 and *Chlorogloeopsis fritschii*) [65,66]. It produces succinate from isocitrate by isocitrate lyase (ICL), and the co-product glyoxylate is combined with an acetyl CoA to generate malate, thus bypassing the decarboxylation steps of the TCA cycle. The GABA pathway can play a key role in cellular tolerance to acidic, heat, and oxidative stresses, while the glyoxylate shunt allows cells to use acetate both as a carbon and energy sources for faster growth [62,66]. 

All three TCA cycle variants (Figure 3) produce succinate, the very important electron donor for oxidative phosphorylation, and offer the metabolic plasticity needed to cope with rapid environmental changes (light and nutrients availabilities, *etc*) [62]. *Synechocystis* PCC 6803, which has only the KGD pathway and the GABA shunt that contribute to the intracellular GABA pool, show that they can be functionally independent of each other. 

## 7. Nitrogen Fixation and Assimilation

Nitrogen is the second-most abundant element in living cells (representing approximately 10% of their dry mass) and is present in amino acids, nucleotides, and cell wall (peptidoglycan) components. In cyanobacteria, nitrogen is accumulated in light-harvesting pigments (chlorophyll, phycocyanin and in many but not all species phycoerythrin) and stored as cyanophycin, a copolymer of arginine and aspartate [67]. Cyanophycin is synthesized by a single enzyme, cyanophycin synthetase, CphA1, in a two-step reaction from L-aspartate and L-arginine, using one ATP per amino acid [36]. Cyanobacteria can use ammonium, nitrate, or nitrite as N sources, and some species can also assimilate urea or amino-acids [24], or fix atmospheric N_2_ [67]. 

Ammonium is the preferred nitrogen source. When it is present with other suitable nitrogen sources, it is utilized first. As ammonium is scarce in most habitats, cyanobacteria possess a high-affinity ammonium uptake system, such as the Amt1 permease of *Synechocystis* PCC 6803. However, ammonium transport must be tightly controlled as high ammonium concentrations can be toxic [68]. 

Cyanobacteria have two types of nitrate/nitrite transport systems, a high-affinity permease NrtP and the ABC-type transporter NrtABCD (NRT). Intracellular nitrate is sequentially reduced to nitrite and then to ammonium by nitrate reductase (NR) and nitrite reductase (NiR), using the ferredoxin-transported electrons provided by photosystem I. Addition of ammonium to nitrate adapted cells triggers an immediate inhibition of nitrate uptake and assimilation. The ammonium-induced inhibition of NRT is regulated by the P_II_ protein and the C-terminal domain of NrtC [68].

For the assimilation of urea, which is frequently present in aquatic ecosytems (it contributes to about 50% of the total nitrogen used by cyanobacteria thriving in oceanic or estuarine waters), cyanobacteria possess a high affinity ABC-type transporter that can import urea even at low concentrations (≤1 μM). This urea transport system, encoded by the five genes cluster *urtABCDE*, is regulated by the NtcA transcription factor [69]. Inside the cells, urea (CO(NH_2_)_2_) is catabolized into ammonia (NH_3_) and CO_2_ by the enzymes urea amidolyase and urease [24]. The ATP-requiring urea amidolyase enzyme has two activities, which can be exhibited by two different proteins: urea carboxylase and allophanate hydrolase in prokaryotes and green algae. The nickel-requiring (ATP-independent) enzyme urease is encoded by seven genes, *ureABCDEFG* (frequently clustered), that compose the urease per se (UreABC) and its Ni-assembly chaperones (UreDEFGH). Recently, we have analyzed the genome of 308 cyanobacteria [24]. We found that most of them harbor all urea transport (*urtABCDE*) and urease (*ureABCDEFG*) genes, indicating that most cyanobacteria should be able to grow on urea as the sole nitrogen source, as observed in the case of the few cyanobacteria already tested [70]. This finding suggests that in the future, the photoproduction of biotechnologically interesting chemicals by engineered cyanobacteria could be coupled to water treatment (urea consumption), as we observed in the case of hydrogen production [70]. Many cyanobacteria possess either *ureABCDEFG* or *urtABCDE*, indicating that some cyanobacteria use their urease to recycle nitrogen and carbon from internally generated urea, while other cyanobacteria employ their *urtABCDE* system to transport various nutrients (and not just urea). Three cyanobacteria of the genera *Gloeobacter* and *Gloeomargarita,* which likely diverged early from other cyanobacteria and lack *ureABCDEFG*, have the genes encoding both urea carboxylase and allophanate hydrolase. These findings suggest that the urea carboxylase and allophanate hydrolase enzymes appeared prior to urease in cyanobacteria [24]. 

Many cyanobacteria can also fix atmospheric dinitrogen (N_2_). As N_2_ is very stable, the fixation of one molecule of N_2_ catalyzed by the nitrogenase enzyme is energetically expensive [67]:

N_2_ +16ATP + 8H^+^ +8e^−^ → 2NH_3_ +H_2_ +16ADP + 16Pi. (2)

Because nitrogenase is irreversibly inactivated by O_2_, the N_2_-fixing (diazotrophic) cyanobacteria developed several strategies to protect their nitrogenase from O_2_. Many diazotrophic filamentous cyanobacteria, such as *Nostoc* sp., respond to nitrogen depletion by differentiating (irreversibly) some of their vegetative cells (one of every 10 to 20 vegetative cells along a filament) into heterocysts [71]. These N_2_-fixing cells harbor a thick extracellular glycolipid envelope that decreases the diffusion of O_2_ and maintains an active respiration that consumes O_2_. Furthermore, heterocysts retain photosystem I to harvest light and produce chemical energy (ATP), but have no O_2_-producing photosystem II [22]. Thus, they cannot generate electrons from water and are dependent on their neighboring vegetative cells for reduced (organic) carbon [67,71]. For example, vegetative cells provide glutamate to heterocysts and receive glutamine from heterocysts [71]. Due to their oxygen-free environment and their genetic tools for heterocyst-specific expression, heterocysts are attracting interest to serve as cell factories for biotechnological purposes [22,72].

The non-heterocystous filamentous (*Lyngbya*) and unicellular cyanobacteria (*Crocosphera*, *Gloeothece* and *Cyanothece*) temporally separate photosynthesis (production of O_2_) during the day from N_2_ fixation during the night, where O_2_ is no longer produced by photosynthesis and is consumed by respiration [67].

Within cells, ammonia is assimilated via the coupled glutamine synthetase (GS) and glutamine-oxoglutarate amidotransferase (GOGAT) reactions. GS catalyzes the ATP-requiring ligation of ammonia with glutamate, producing glutamine. The subsequent GOGAT reaction transfers the amido-group of glutamine to the α-C atom of 2-oxoglutarate (2OG), yielding two molecules of glutamate. The net yield of the GS–GOGAT cycle is therefore the conversion of 2OG with ammonia into glutamate, consuming one ATP and two reduction equivalents [73]. Then, the organic nitrogen of glutamate and glutamine are distributed to the other amino acids and various cellular building blocks, in a metabolic architecture placing 2OG at the intersection of carbon- and nitrogen-assimilatory reactions [36].

To avoid a detrimental glutamate depletion, the activity of GS must be tightly adjusted to the glutamate-refilling GOGAT activity. GS activity is mainly regulated by the small inhibitory proteins IF7 and IF17, encoded by the *gifA* and *gifB* genes in *Synechocystis* PCC 6803 [74]. Considering the 2OG decarboxylase reaction (see below) as a minor bypass, the flux from 2OG to glutamate, catalyzed by GOGAT, represents the dominant metabolic route for the consumption of 2OG among cyanobacteria [73].

## 8. Coordination of the Carbon and Nitrogen Metabolisms

Carbon and nitrogen are the two most abundant nutrient elements for all living organisms. In cyanobacteria the processes of nitrogen and carbon assimilation are strongly interdependent in competing for electrons (NAD(P)H) and ATP provided by photosynthesis [75]. Consequently, cyanobacteria have evolved a sophisticated regulatory system to maintain a carbon to nitrogen ratio of about five to one, which implies that for five molecules of CO_2_ one molecule of ammonia must be assimilated [36]. This regulation system involves a complex signal transduction network employing three types of actors: (i) the transcription factors NdhR [76], CmpR [36], NtcA [69], LexA [77,78], and AbrB2 [76]; (ii) the key metabolites 2-oxoglutarate (2OG) [73] and 2-phosphoglycolate (2PG); and (iii) the regulatory proteins P_II_ [68] and PipX [36].

Recently, several non-coding RNAs have been identified that play crucial roles in the regulation of the carbon and nitrogen metabolisms and their crosstalk [79].

### 8.1. Role of Key Transcription Regulators (NdhR, CmpR, AbrB2, and LexA) and Associated Key Metabolites (2-oxoglutarate and 2-phosphoglycolate)

In cyanobacteria, three LysR-type transcriptional factors, NdhR [80], CmpR [81], and AbrB2 [82], operate in the regulation of the CCM [36]. The CCM system is expressed at a basal level under CO_2_-replete conditions whereas its expression and activity are strongly enhanced under Ci limitation. The transcription factors NdhR and CmpR, which share a high sequence identity [76], are regulated by the small metabolites 2OG (2-oxoglutarate) and 2PG (2-phosphoglycolate). The 2OG metabolite, the carbon skeleton for nitrogen assimilation [73], is produced from CO_2_ fixation products via glycolysis and the TCA cycle (via the isocitrate dehydrogenase), and is consumed by the nitrogen assimilating GOGAT reaction (linked to GS activity). Under nitrogen limitation, when ammonia assimilation via GS cannot provide enough glutamine for the subsequent GOGAT reaction, 2OG consumption is slowed down and its levels are increased. The accumulation of intracellular 2OG is an indicator of a high C/N ratio. In contrast, the accumulation of 2PG, generated by the oxygenase activity of RubisCO, is an indicator of a low C/N ratio [36,76].

#### 8.1.1. Role of the NdhR Transcription Factor 

**Figure 4:**
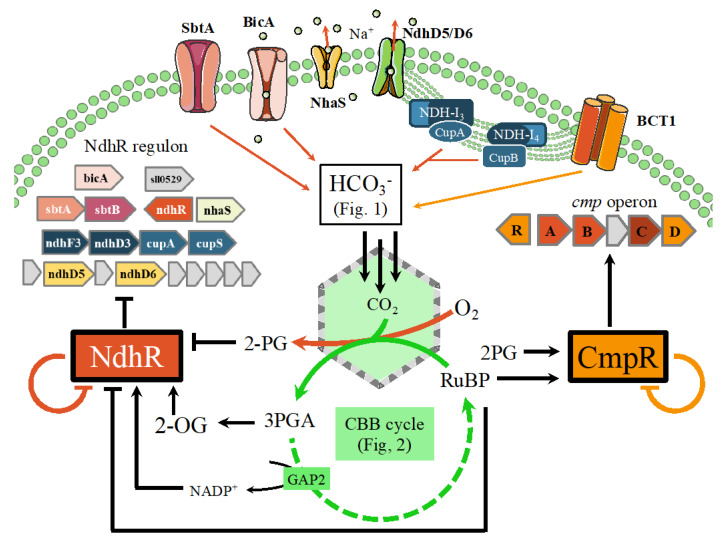
Regulation of the *Synechocystis* PCC 6803 carbon concentration mechanism by the CmpR and NdhR transcription factors and their modulation by the NADP^+^, 2OG (2-oxoglutarate, an indicator of high C/N ratio), 2PG (2-phosphoglycolate, an indicator of low C/N ratio), and RuBP metabolites. NADP^+^ and 2OG produced by the Calvin cycle accumulate when Ci is abundant. They stabilize the NdhR tetramers, favoring its repressor activity on the promoters of target genes encoding BicA (*sll0834*), SbtAB (*slr1512*-*slr1513*), NhaS (*slr1727*), NdhD3-F3-CupA-CupS (*sll1732*-s*ll1733*-*sll1734*-*sll1735*), and NdhD5-D6 (*slr2007*-*slr2009*), as well as its own gene (*sll1594*) [76,80,83]. The *sll0529* gene encodes a putative transketolase whose function is not known. Under low Ci conditions, the increased O_2_/CO_2_ ratio leads to the oxygenation of RuBP that produce 2PG, which competes with 2OG for binding on NdhR, ultimately leading to its release from its target promoters. In addition, 2PG acts as a positive effector of CmpR that stimulates expression of the bicarbonate transporter BCT1, encoded by the *cmpABCD* operon (*slr0040*-*slr0041*-*slr0042*-*slr0043*-*slr0044*), while repressing its own promoter (*sll0030*) in opposition to that of the *cmpABCD* operon. Its affinity for the target DNA sequence is increased by RuBP that is also accumulated because of the lower activity of the Calvin cycle.

The NAD(P)H dehydrogenase transcription factor (NdhR), a global repressor of carbon-assimilation genes (Figure 4), was originally identified by us [80] as the repressor of its own gene expression as well as of the *ndhF3*/*ndhD3*/*cupA*/*sll1735* gene cluster (the *ndh-I3* operon), which encode the high-affinity CO_2_-uptake system proteins [76]. NdhR (also called CcmR [83]) represses the genes encoding the two sodium-dependent bicarbonate uptake systems: SbtA (a high affinity/low flux transporter) and BicA (a medium affinity/high flux transporter), using, as co-repressors, the 2OG and NADP^+^ metabolites that are both accumulated under CO_2_-sufficient conditions [76]. NdhR binds as a tetramer to the promoter regions of its target genes thereby blocking their transcription. The binding of 2OG onto NdhR stabilizes its tetrameric conformation with its high DNA binding affinity [36]. This effect is antagonized by 2PG that binds onto a different NdhR regulatory site, which then dissociates from its target gene operators, allowing transcription of the CCM genes and leading to increased carbon input. The binding of 2OG or 2PG to NdhR being mutually exclusive, their relative levels determine whether NdhR acts as a repressor or not, to maintain the C/N balance. When 2OG levels increase due to carbon excess or nitrogen deficiency, CCM activity is repressed to its basal level. In contrast, at high levels of 2PG the CCM-related genes are derepressed to increase the CCM-dependent CO_2_ fixation by RubisCO [36].

#### 8.1.2. Role of the CmpR Transcription Factor

CmpR activates the *cmpABCD* operon (Figure 4), which encodes an ABC-type bicarbonate transporter (BCT1), whereas it represses its own gene. Its DNA binding affinity is increased by the addition of the metabolites RuBP or 2PG, whose concentrations are expected to increase under low CO_2_ conditions. The crystal structure of the *Synechococcus* PCC 7942 CmpR was solved in a complex with RuBP, indicating that RuBP-induced conformational changes of CmpR to affect the regulation of the *Cmp* operon [36].

Considering the 54% sequence identity with NdhR, it has been proposed that CmpR binds to 2PG (produced under carbon limitation) in a manner similar to 2PG binding by NdhR, although CmpR and NdhR regulate gene expression in an opposite manner. The opposite effects of 2OG and 2PG on the activity of NdhR provide an example of the integration of the metabolic signaling necessary for cyanobacterial adaptation in response to the cellular status of carbon or nitrogen metabolism [76].

#### 8.1.3. Roles of the AbrB2 Transcription Factor

In addition to NdhR and CmpR [76], the AbrB2 transcription factor, which represses the hydrogenase operon [84], was found to positively regulate CCM-related genes (*cmpABCB*, *sbtAB* and *ndhF3/ndhD3/cupA* operons) under carbon limitations [85]. Intriguingly, AbrB2 appeared to positively regulate several genes of the NtcA-regulon, including *urtA*, *amt1*, *glnB*, *sigE* and the *nrtABCD* operon [36]. In contrast, we found no AbrB2-mediated regulation of the *urtA*, *amt1*, *glnB* genes and the *nrtABCD* operon [86]. This discrepancy might somehow result from the fact that our unlike AbrB2-deleted mutant (constructed in the true wild-type background) grows as healthily as the WT strain, whereas the AbrB2-deleted mutant constructed by Ishii and co-workers (in a glucose-tolerant genetic background) exhibits a slow growth [82].

#### 8.1.4. Roles of the LexA Transcription Factor 

In *Synechocystis* PCC 6803, we showed that the LexA transcription factor does not regulate the genes involved in DNA repair, unlike the well-known LexA (SOS) regulator of *E. coli* [77]. Instead, the *Synechocystis* PCC 6803 LexA protein directly regulates numerous genes operating in carbon assimilation or controlled by carbon availability [77], including the bidirectional hydrogenase [87,88] and the RNA helicase CrhR [89]. Recently, LexA was shown to directly bind to the promoter regions of genes involved in twitching motility, and biosynthesis of fatty acids and the major compatible solute, glucosyl-glycerol [78].

### 8.2. Roles of the Key Transcription Factor NtcA and the Regulatory Proteins P_II_, PipX

Expression of the nitrogen assimilation genes encoding GS, GOGAT, and GS-inhibiting proteins, GifA and GifB, is controlled by: (i) the transcription factor NtcA [69], (ii) the P_II_ and PipX proteins [68], (iii) the nsiR4 non-coding RNA, and (iv) a glutamine riboswitch in the 5′UTR of *gifB* [36]. 

NtcA, a transcription factor belonging to the CRP (cAMP receptor protein) family serves as the global regulator for nitrogen assimilation and metabolism. Of biotechnological interest, NtcA has been shown to control the expression of microcystin (a toxin) synthesis genes in *Microcystis aeruginosa* PCC 7806 [90], as well as the production of ethylene in an engineered strain of *Synechocystis* PCC 6803 [91]. The P_II_ protein indirectly regulates NtcA through binding to the NtcA co-activator PipX (P_II_ interacting protein X). P_II_ senses both the energy status of the cell by the competitive binding of ADP *vs* ATP, and the carbon/nitrogen status by the binding of 2OG. In addition, depending on nitrogen availability, P_II_ can be phosphorylated [68].

Under nitrogen limitation (high 2OG levels), NtcA activates the expression of genes for nitrogen uptake and assimilation, including *urtA, nirA, ntcB and glnA*, and it represses the *gifA* and *gifB* genes encoding the GS inactivating factors IF7 and IF17. Furthermore, the accumulation of 2OG stimulates the binding of NtcA to its target genes. Maximal activation of NtcA requires the subsequent binding of the coactivator PipX. Under conditions of low nitrogen abundance, P_II_ binds 2OG in a cooperative manner with adenosine triphosphate (ATP) and is phosphorylated. This causes the release of PipX and its interaction with NtcA, stabilizing the active 2OG-bound conformation of NtcA. Recently, it was shown in *Synechocystis* PCC 6803 that PII can form a specific P_II_ -PEPC complex that influences the activity of PEPC (phosphoenolpyruvate carboxylase), the second major carbon-fixing enzyme in photoautotrophic organisms (see above). Whereas in the absence of P_II_, PEPC is subjected to ATP inhibition, it is activated beyond its basal activity in the presence of P_II_. Furthermore, P_II_-PEPC complex formation is inhibited by ADP and PEPC activation by P_II_-ATP is mitigated in the presence of 2-OG, linking PEPC regulation to the cell’s global carbon/nitrogen status [92]. 

When nitrogen is abundant, P_II_ binds to PipX to counteract NtcA activity [69,75]. P_II_ also interacts with the Amt1 ammonium permease, the NrtC and NrtD subunits of the nitrate/nitrite transporter NrtABCD, and the UrtE subunit of the ABC-type urea transporter UrtABCDE. The deregulation of urea uptake in a P_II_ deletion mutant causes ammonium excretion when urea is provided as nitrogen source. Furthermore, P_II_ regulates arginine biosynthesis by interacting with the rate-limiting enzyme *N*-acetylglutamate kinase (NAGK) [93]. If sufficient energy and nitrogen are available, indicated by a high intracellular ATP and low 2OG levels, non-phosphorylated P_II_ interacts with NAGK, enhancing its catalytic efficiency and relieving it from feedback inhibition by arginine. At high intracellular arginine levels, the carbon/nitrogen storage polymer cyanophycin (multi-L-arginyl-poly-L-aspartate) accumulates in *Synechocystis* PCC 6803 [67]. In addition, P_II_ protein can control acetyl-CoA levels by interacting with the biotin carboxyl carrier protein (BCCP) of acetyl-CoA carboxylase (ACC) [68]. Furthermore, other status reporter metabolites have recently been discovered, including cAMP that is sensed by the P_II_-like protein SbtB [36].

Intriguingly, a recent study showed that reactive oxygen species (ROS) naturally generated by respiration and photosynthesis interferes with C/N status sensing by decreasing the intracellular concentrations of 2OG, thereby altering the function of the nitrogen regulator NtcA [73,76]. Furthermore, the 2OG-sensing global signal detected by NtcA is combined with redox-stress signal perceived by the Hik33/RpaB relay [73]. 

## 9. Redox Regulation of the Central Metabolism

Reversible protein thiol oxidation is an essential post-translational regulatory mechanism enabling living organisms to respond to changes in metabolic demands and environmental conditions. In *Synechocystis* PCC 6803, the redox dynamics of ~2100 cysteinyl amino acids sites from 1060 proteins under light, dark, and 3-(3,4-dichlorophenyl)-1,1-dimethylurea (DCMU, a photosystem II inhibitor) conditions were quantified. The overall results revealed broad changes in thiol oxidation in many key biological processes, including photosynthetic electron transport, carbon fixation, and glycolysis [94]. 

A group of well-known redox-sensitive proteins, thioredoxins, play a major role in the regulation of cellular processes in cyanobacteria [95]. The thioredoxin-mediated regulation involves the post-translational modification of cysteinyl residues on target proteins, reducing disulfide bridges to thiol groups [96]. 

In higher plants, two thioredoxins (Trx f and Trx m) were first identified as activators of enzymes involved in photosynthetic carbon assimilation in the chloroplast. The Trx redox state links the activity of the Calvin cycle enzymes phosphoribulokinase (PRK), NADP-glyceraldehyde-3-phosphate dehydrogenase (GAPDH), fructose 1, 6-bisphosphatase (FBP), and sedoheptulose 1,7-bisphosphatase (SBP) to the supply of ATP and NADPH in response to variations in light intensity [96].

### 9.1. Prominent Role of the Redox-Responsive CP12 Protein

A small intrinsically-disordered protein, CP12, operates in the thioredoxin-mediated regulation of the Calvin cycle, in mediating the formation of a complex between glyceraldehyde-3-phosphate dehydrogenase (GAPDH) and phosphoribulokinase (PRK) in response to changes in light intensity [96,97]. Under low light, the formation of the PRK/CP12/GAPDH complex results in a reduction in the activity of both PRK and GAPDH. Under light conditions, a thioredoxin mediates the dissociation of the complex to increase both GAPDH and PRK activity [96]. The formation/dissociation of the PRK/CP12/GAPDH complex provides a rapid regulation of the rate of carbon fixation by the Calvin cycle in response to changes in light availability to produce NADPH and ATP [98]. In pea leaves exposed to high light, dissociation occurred in under 1 min, while re-association was evident 1 min after transfer to low light and complete after 5 min under darkness [96].

A number of studies suggest that CP12 proteins may play a wider role [96,97,99]. In *Arabidopsis thaliana* CP12 is expressed in a range of tissues, including the roots which usually receive no light. Furthermore, similarly to higher plant genomes that encode up to three forms of CP12, cyanobacterial genomes possess multiple CP12 genes, raising questions about the role of these different CP12 proteins [97]. The diverse primary structure of cyanobacterial CP12-like proteins can be classified into eight different groups based on the presence or absence of the three conserved features of classical CP12 proteins, i.e., the N- and C- terminal cysteine pairs and the central highly conserved “AWD_VEE” motif [97]. When oxidized, the N- and C-terminal cysteine pairs form two intramolecular disulfide bridges, which are necessary for the formation of the PRK/CP12/GAPDH complex, and are converted to thiol groups when reduced by Trx. On conversion to the reduced form, CP12 loses the conserved α-helices present and becomes completely unstructured, flexible, and inactive [97]. 

The discovery of eight CP12-like cyanobacterial classes, some of whom have proteins that are fused to an N-terminal cystathionine-β-synthase (CBS) domain, raises interesting questions about the role of these fusion proteins in the regulation of metabolism [96]. No individual cyanobacteria has all eight different classes of CP12-like proteins, but, except for the marine picocyanobacteria, all other groups possess at least one copy of the classical CP12 form [96].

Metal binding studies in vitro have shown that the *Chlamydomonas* CP12 protein can bind copper (Cu^2 +^) with an affinity (Kd 26 μM) similar to that of the prion protein (Kd of about 14 μM) and nickel-binding chaperone proteins (Ni^2+^ Kd 11 μM) [96]. There is evidence showing that Cu^2+^ ions aid the formation of disulfide bonds in the *Chlamydomonas* CP12 protein, though this protein can interact with GAPDH and PRK in the presence or absence of copper ions [96]. In addition, the backbone structures of the *Synechococcus* PCC 7942 CP12-GAPDH binary complex in copper-free and copper-bound forms are very similar, suggesting that copper is not essential for the formation of the PRK/CP12/GAPDH complex [96].

In *Synechococcus* PCC 7942, the analysis of a CP12 knockout mutant indicated that CP12 operates in the separation of the activities of the Calvin cycle from the oxidative pentose phosphate pathway (OPP) during day-night cycles. Interestingly, some cyanobacterial phages exploit this regulatory mechanism by introducing a CP12-like protein into the cyanobacterial host to downregulate its Calvin cycle and upregulate its OPP [100]. Furthermore, phage genes involved in OPP, photosynthesis, and deoxynucleotide biosynthesis are expressed in the host cyanobacteria, likely to boost the production of NADPH and deoxynucleotides for phage replication [96]. 

Of biotechnological interest, it has been shown in a *Synechococcus* PCC 7942 strain engineered for the production of 2,3-butanediol that the level of this production was increased by the deletion of the CP12 gene combined with the overexpression of the RubisCO operon (*rbcLXS*) and the phosphoribulokinase (*prk*) gene [101].

### 9.2. Glutathionylation: An Overlooked Mechanism Operating in the Regulation of the Central Metabolism

**Figure 5:**
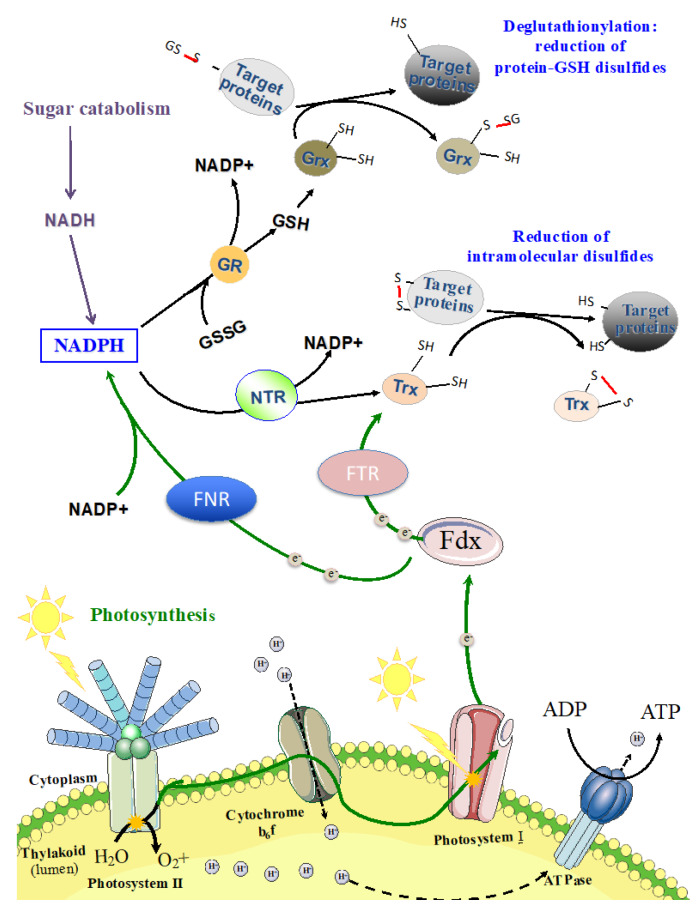
Schematic representation of the redox regulation mechanisms. Fed: ferredoxin; FNR: ferredoxin NADP+ reductase; FTR: ferredoxin thioredoxin reductase; GR: glutathione reductase; Grx: glutaredoxin; GSH: reduced glutathione (monomer form); GSSG: oxidized glutathione (glutathione disulphide = dimeric form); H^+^: proton; NTR: NADPH dependent thioredoxin reductase; SG: glutathionylated cysteine; SH: thiol in a cysteine residue; Trx: Thioredoxin. The green lines represent the photosynthetic electron transfer, while the purple lines correspond to the sugar catabolism.

Photosynthetic organisms are continuously challenged with toxic reactive oxygen species (ROS) generated mostly by photosynthesis and respiration. These ROS are handled by cellular defense systems that, among other processes, maintain the redox homeostasis of cellular thiols (Figure 5). Glutathione, a very important player in this process, is a highly abundant (from 0.1 to 10 mM) tripeptide (γ-Glu-Cys-Gly) that occurs in two forms. The reduced (major) form (GSH) maintains the intracellular cell environment in a reduced state. The resulting glutathione disulfide (GSSG) is reduced back to GSH by various factors, such as the NADPH-using enzyme glutathione reductase (GR) that occurs in most cyanobacteria, with the noticeable exception of the two well-studied models *Synechocystis* PCC 6803 [102] and *Synechococcus* PCC 7002 [103]. 

Glutathione can form a mixed-disulfide bridge between the thiol group of its cysteine and an accessible thiol of a protein (Figure 5). This reaction, called protein glutathionylation, can occur spontaneously by reacting with activated thiols (sulfenic acids) or it can be catalyzed by specific enzymes, such as glutathione transferase (GST) [104]. As this post-translational modification is reversible, it constitutes an important mechanism of redox signaling by protecting specific cysteine residues and/or by modulating protein activity [105,106]. The reverse reaction, deglutathionylation (Figure 5), is catalyzed by the thiol−disulfide oxidoreductase thioredoxins (Trx) or the GSH-dependent enzymes GST or glutaredoxins (Grx) [106].

Using *Synechocystis* PCC 6803 as a model, we recently identified 383 proteins that can be glutathionylated *in vitro* [107]. These glutathionylable proteins participate in a wide range of cellular processes and metabolic pathways, including carbon and nitrogen metabolisms (see below), cell division, stress responses, and hydrogen production. The glutathionylation of several proteins, namely, the antioxidant peroxiredoxin II, the mercuric reductase, the AbrB2 transcription factor, and the metabolic enzyme 3-phosphoglycerate dehydrogenase, was confirmed by biochemical studies of the purified recombinant proteins [105,106,107]. 

Nine proteins operating in the Calvin cycle were identified as potential targets of glutathionylation, namely: RubisCO (small and large subunits), phosphoglycerate kinase (PGK), D-fructose 1,6-bisphosphatase class 2/sedoheptulose 1,7-bisphosphatase, transketolase, transaldolase, ribulose-phosphate epimerase, glyceraldehyde-3-P dehydrogenase (GAPDH), phosphoribulokinase (PRK), and CP12 [107]. The possible glutathionylation of the GAPDH, PRK, and CP12 proteins is of particular interest since it could influence the formation of the PRK/CP12/GAPDH supramolecular complex that down-regulates GAPDH and PRK activities [107]. Together, these data suggest that glutathionylation could regulate the Calvin cycle in cyanobacteria facing oxidative stress [107] as observed in photosynthetic eukaryotes [108]. Again as observed in eukaryotes, two sugar metabolism enzymes were identified as glutathionylable [107,108].

Furthermore, numerous putative targets of glutathionylation are involved in nitrogen assimilation and amino-acid metabolism, such as glutamine synthetase, GOGAT, methionine synthase, and acetolactate synthase, which operates in the synthesis of leucine and isoleucine as well as in the TCA cycle that produces NADPH and carbon metabolites for nitrogen assimilation and thereby cell growth [36,62]. 

Collectively, these results suggest that glutathionylation constitutes a major mechanism of global regulation of the cyanobacterial metabolism under (photo)oxidative stress conditions that can occur especially in large-scale cultures for biotechnological projects [107].

## 10. Cyanobacteria Facing Carbon/Nitrogen Imbalance can Produce Exopolysaccharides and Biodegradable Plastics of Biotechnological Interest

In response to nitrogen limitation, many cyanobacteria can increase their production of extracellular polysaccharides (EPS), which harbor six to twelve types of monosaccharides, are more complex than the EPS formed by other bacteria or eukaryotic microalgae, which usually contain less than four monosaccharides [109]. Cyanobacterial EPS are usually strongly anionic since they contain one or two different uronic acids, as well as sulfate groups, a rare feature among bacteria [109]. The presence of negatively charged EPS surrounding cyanobacterial cells may play an important role in adsorption sequestration of metabolites, inorganic micronutrients, and/or metal cations [110,111], creating an extracellular pool of resources that can be re-used [112]. The EPS can also serve as the structural scaffold for the formation and maintenance of biofilms, which protect internal cells against various noxious agents, including O_2_, UV, salt, and heavy metals [110,113], eventually combine to result in the sacrificial death of surface-exposed cells. Hence, EPS can be of biotechnological interest for processes such as the adsorption of toxic metals for water treatment and/or controlling cell buoyancy (sedimentation/flotation) to facilitate biomass harvesting [113,114]. Furthermore, cyanobacterial EPS also have the potential to serve as food-thickening agents [115] or in wound-healing or anti-inflammatory treatments [116]. Cyanobacterial EPS are also involved in the colonization of soils and the biomineralization of calcium (and/or magnesium) carbonates, sometimes leading to the formation of stromatolites [6]. 

Cyanobacteria facing nitrogen starvation can also produce poly-3-hydroxybutyrate (PHB) [117], the best-characterized member of the polyhydroxyalkanoates (PHAs) family of biodegradable plastics [118], as carbon storage compounds [119]. These PHB form water insoluble inclusions (granules) inside the cyanobacterial cells that can make up between 5–20% of the dry cell weight [120,121]. The accumulation of PHB is highly heterogeneous at the single-cell level [122]. The main carbon flux in the light-driven CO_2_-capturing synthesis of PHB goes from the Calvin cycle, through the lower part of glycolysis, to pyruvate and then acetyl coenzyme A (acetyl-CoA). Then, two acetyl-CoA groups are condensed into aceto-acetyl-CoA and subsequently reduced by NADPH to hydroxybutyryl-CoA which can then be polymerized to PHB [123].

The CO_2_-capturing photosynthetic production of PHA/PHB polymers by cyanobacteria is very interesting since these bioplastics can be completely degraded into CO_2_ and water by naturally occurring microorganisms [118]. In contrast, the petroleum-based plastics massively used in packaging industries are refractory to biodegradation. Thus, they accumulate as microplastics in oceans [124] and challenge the production and quality of the marine food chain (production of plankton, entanglement or intestinal blockage or gastric impaction of fishes *etc*) [125].

## 11. Concluding Remarks

Cyanobacteria are widely diverse photosynthetic organisms that play a crucial role in the biosphere, including the production a large part of the organic matter and oxygen for the rest of the food chain. They also have a great potential for the production of chemicals from solar energy, fresh or marine water, and minerals, thanks to their powerful photosynthesis and the effective synthetic biology tools of several model strains [23]. To turn the biotechnological promise of cyanobacteria into an industrial reality there are several options. We can continue to study a few genetically tractable species (such as *Synechocystis* PCC 6803, *Synechococcus* PCC 7942, *Synechococcus* PCC 7002, and *Anabaena* PCC 7120) to continue to identify key metabolic players (enzymes and protein regulators and their co-factors; RNA regulators, metabolites, *etc*.). When this is achieved, mathematical models will be of great help to design genetic engineering strategies of these model hosts for the photosynthetic production of biotechnologically important chemicals. However, one cannot guarantee that it is possible to combine in the same genetic model all properties required for the efficient and stable photoproduction of an intended chemical. Knowing that the large biodiversity of cyanobacteria has been frequently overlooked so far, it is certainly interesting to begin studying non-model cyanobacteria with the goal of combining their specific interest with the engineering strategies developed in well-studied genetic model. To do so major challenge remains to determine if, and when, the required genetic toolbox developed for the well-studied cyanobacterial models can be adapted to these "new" model cyanobacteria emerging from such investigations. Furthermore, communication should be strengthened between academic researchers who engineer cyanobacteria for biotechnological purposes, but have a limited access to large-scale photobioreactors, and industrial partners who attempt to use the engineered cyanobacteria to actually produce intended chemicals at reasonable costs, but may lack academic knowledge on cyanobacterial physiology and metabolism. Finally, it would certainly be helpful to put a tax on the CO_2_ emitted by oil combustion and transformation, to be used to decrease the cost of the photosynthetic production of chemicals by cyanobacteria in order to turn their promises into industrial realities.
